# The Poise of Scholarship1Address delivered to the graduating class of St. Margaret’s School, June 9, 1909.

**Published:** 1909-08

**Authors:** Henry Reed Hopkins

**Affiliations:** 97th President of the Medical Society of the State of New York; Honorary Life Member of the Medical Society of the County of Erie


### BUFFALO MEDICAL JOURNAL.

VOL. LXV. AUGUST, 1909. No. 1

### ORIGINAL COMMUNICATIONS.

### The Poise of Scholarship.[1]

BY HENRY REED HOPKINS, M.D.

97th President of the Medical Society of the State of New York.

Honorary Life Member of the Medical Society of the County of Erie.

"THE more we study the body and the mind, the more we find both to be governed, not by, but according to laws such as we observe in the larger universe."

I once heard an eloquent speaker close an address to a world's congress with these words: "I have a mortal body, I have a human mind, I am a living spirit."

These thoughts may help to prepare our minds for the consideration of our topic "The Poise of Scholarship."

You will, I trust, grant me the indulgence of a moment's digression, and allow me to declare and make unmistakable that the scholarship we are considering, the scholarship of your faculty of teachers, of your distinguished principal, of your Right Reverend Father, Bishop and Visitor, the scholarship of St. Margaret's school, is Christian scholarship; that Christian scholarship assumes the existence of the universe, of the heavens and of the earth, to be the work of an all wise personal Creator; predicates man as a person whose personality is manifested by the characteristics thought, will and love; that man's personality is not a disconnected aggregate, but an indivisible organic whole; his thoughts the thoughts of one who wills and loves; his will that of one who thinks and loves; his love that of one who thinks and wills.

Christian scholarship further assumes the being and personality of God—the God of the creeds of the Catholic Church and the existence of sin and man's need of redemption.

This particularity of statement is necessary in order to avoid ambiguity, uncertainty and weakness, as there is that which uses the name of scholarship, even the most advanced and learned

1. Address delivered to the graduating class of St. Margaret's School, June 9, 1909.

scholarship, and yet ignores or denies each and every one of the foregoing affirmations.

The poise of scholarship may now engage our attention and our thought will advance towards definiteness of conception by recalling what poise is not.

Poise is not breadth, largeness, amplitude; not height, superiority, exclusiveness; not even moderation, sweetness and light. No! Desirable, delightful and greatly to be prized as are these qualities we must admit that poise is none of them. Poise is a just and accurate recognition of relationships, an intelligent appreciation of values, even when values are in appearance or in reality antithetic.

A single illustration will help to make my meaning clear: mathematics and astronomy teach us that the earth, the planet on which we live, like the other heavenly bodies, the planets of our system, is in constant motion revolving around the sun; that this motion of revolution is caused by the force called the attraction of gravitation, a property common to all matter; the force which gives matter this quality we know as weight; a force which operates between all bodies in proportion to their relative weights and distances. Astronomy further teaches us that our sun is 1,384,472 times larger than the earth, and is also 354,936 times heavier, and that the earth, in its orbit around the sun travels at the rate of 68,040 miles an hour.

When we recall that the orbital motion of the earth around the sun is the result of the attraction of one globe for the other, and then further remember the enormous disparity in size and weight of the sun and the earth, and the frightful speed at which the earth moves we are quite ready to forgive the mistake of the early astronomers who believed and taught that the path of the earth about the sun was a circle.

However, knowledge of mathematics, of astronomy, of physics grew; Newton and Kepler came and spoke to the earth and were taught knowledge; spoke to the heavens and were taught wisdom, and in the light of this knowledge saw and declared that the path of the earth about the sun was not a circle but an ellipse; that notwithstanding the enormous comparative size and weight of the center of our system, the sun—that the earth was so made by the All-wise Creator that it had poise, and that the poise of the earth compelled it to recognise the weights and positions of the several planets of our system, that the poise of the earth compelled it to leave the circular portion of its orbit—that nearest to the sun—for the almost straight line of the ellipse, and the same poise again compelled the earth to leave the lesser curve for the greater one of the circle, and again to change its course and to speed in an almost straight line towards the sun, the poise of the earth thus making its yearly path about the sun to consist of alternating motions four in number, first, a sharp curve of November to February, a lesser curve from February to June, then a sharp curve from June to August and again a lesser curve from August to November.

The degree of the ellipticity of the earth's orbit is indicated by the fact that at this moment we are more than three millions of miles farther from the sun than during the month of January when at the other end of the ellipse.

It should open our minds to the significance and importance of poise to recall that our seasons, summer and winter, the variations of day and night, and in all probability the possibility of life as we know it upon the earth, we owe to the fact that the poise of the earth gives us an ellipse instead of a circle.

We may now with greater advantage hear again the words of Oliver Wendell Holmes—"The more we study the body and the mind, the more we find both to be governed, not by, but according to laws such as we observe in the larger universe."

To my mind it is not by accident but by wise provision that our mental, moral and religious lives are so conditioned that we are constantly and incessantly called upon to choose between two alternatives—the easy motion of the circle about a given concept or the more difficult execution of the ellipse around two focal points. That as in the physical world we have light and darkness, heat and cold, matter and force, attraction and repulsion, growth and decay, life and death, motion and rest, toil and repose, summer and winter, sleeping and waking, feasting and fasting, ebb and flow, youth and age, body and soul; even so in the moral and religious worlds there are the energising concepts of God and man, worship and sin, man and Satan, love and hate, right and wrong, good and evil, life and death, growth and decay, joy and sorrow, temptation and self-control, knowledge and ignorance, power and helplessness, nature and grace, knowledge and faith, belief and conduct.

Constantly and incessantly in our mental exercises, in our practical affairs and in our religious experiences we are invited, nay, more—we are compelled to the choice, shall it be the circle or shall it be the ellipse? A single illustration from each of these categories must suffice: By the common consent of the scholars of all times philosophy has held an honorable position among the nobler, the more exalted of the mental activities. By the same consent of the scholars of today, materialism, naturalism, positivism, pantheism are the more attractive, the more plausible, the more popular chapters of philosophy. And yet Christian scholarship amply demonstrates that the claims and conclusions of materialistic, pantheistic philosophy are only reached and supported by reasoning which is circular where it should be elliptical, by false reasoning which ignores, minimises or denies the possibility of the existence in man of free will—the power of self-control, which reduces personality to the level of the phenomena of our flowers and fruits, which degrades and animalises our notions of the mind, denies the existence of spirit apart from the functions and forces of matter and which makes religion and worship a paradox and a mockery.

Again we read in our well reputed magazines or we hear from our popular pulpits, or even from a college professor or president, popular sentiments to the effect that religion is much too narrow; that there is too much talk about the creeds; that knowledge, not faith; conduct, not belief is what the world now needs; and men are recruited for works of charity or philanthropy—to brotherhood work and to work in social settlements of small and scant religious convictions and not infrequently apparently to prove that the fewer religious convictions a man has the better, more practical worker he is likely to become. What is this but lack of poise leading to a virtual denial of one of the most important practical truths of history—namely, that faith which blesses man with the vision of God, that belief in God, love of God, does lead men the better to love their fellow men; that the man is the better worker for man who loves the whole man, body, mind and soul, and that because he believes his brother was made in the image of God, and that for his salvation our Lord was crucified. What is this but the recognition that two are more than one, that the ellipse is greater than the circle—that poise is essential to practical life?

We will conclude with an illustration from a modern and popular religious creed in which lack of poise seems to be conspicuous, and exercise of denial fully developed. This creed includes the following affirmations. "God is truth, love, mind; God is all; and that all may see that the mental action in this concept is to be circular and by no possibility elliptical proceeds further to affirm—"There is no sin. There is no disease. There is no matter. There is no death. It may aid us here to recall that the criticism of scholarship upon the philosophy of materialism and pantheism is to the effect that materialistic and pantheistic implications and inferences are antagonistic to the higher and nobler conceptions of mind, will, moral sense, self-control, spirit, personality, and that to a degree which makes them distinctly disintegrating and destructive; that poise in philosophical considerations is an attainment sometimes difficult and always important and that philosophy without poise has no place in progressive constructive scholarship.

If poise is desirable in philosophy and expedient in practical life, in religious life it is indispensable, as seems to be sufficiently demonstrated in our illustration of a religious creed including a categorical denial of the existence of matter, sin, disease and death. As does the intellectual world, so the material world offers for our study and inspiration innumerable instances of the vital and practical importance of poise.

The poise of the earth around the sun may well be mentioned first, like unto it in beauty, in suggestiveness, in inspiration, I would cite the poise of the flying eagle—the soaring of the eagle, the bird of freedom—the flying, soaring, poising eagle, the emblem of the ideals, the aspirations of the American people.

In sunshine, in cloud and darkness, when tempests rage, we may well remember the words of the Hebrew scholar, "They who wait upon the Lord shall mount up with wings as eagles.

433 Franklin Street.

